# Transvenous Extraction and Removal of Pacing Leads Placed after Cardiac Transplantation

**DOI:** 10.1155/2019/6270950

**Published:** 2019-01-03

**Authors:** Caleb Norton, Benjamin Holmes, Asad Al Aboud, Eun-Jeong Kim, Holly Gonzales, Christopher Ellis, Roy John, George H. Crossley, Jay Montgomery

**Affiliations:** ^1^Department of Medicine, Vanderbilt University Medical Center, Nashville, TN, USA; ^2^Division of Cardiovascular Medicine, Vanderbilt University Medical Center, Nashville, TN, USA

## Abstract

There is an increasing prevalence of cardiac implantable electronic devices (CIEDs) due to expanding adoption and availability of these evidence-based therapies. With the increased prevalence of these life-saving devices, there has also been an increased demand for lead removal and lead extraction. Understanding the specific subgroups of patients at high risk for complications during and after lead extraction has become imperative to properly manage endovascular CIED leads. There have been multiple published studies describing clinical variables that predict adverse outcomes in CIED system extractions; however, the risk of complications in leads placed after cardiac transplantation has not specifically been addressed to date. We present four cases of transvenous extraction and removal of pacing leads placed after cardiac transplantation. There were no major complications related to extraction in these four cases; however, three of the four patients died within one year after the procedure. While the etiology of death in these cases seemed to be unrelated to the extraction procedure, the indications for extraction (infection in the setting of immunosuppression and calcineurin-associated ESRD and poor sensing/capture possibly secondary to chronic rejection and/or frequent right heart biopsies) likely contributed at least indirectly to the subsequent death.

## 1. Introduction

There is an increasing prevalence of cardiac implantable electronic devices (CIEDs) due to expanding adoption and availability of these evidence-based therapies [1–3]. From 1993 to 2006, it is estimated that index pacemaker and implantable cardioverter-defibrillator (ICD) implants in the U.S. increased from 46.7 to 61.6 and 6.1 to 46.2 per 100,000, respectively [4]. With the increased prevalence of these life-saving devices, there has also been an increased demand for lead removal and lead extraction (defined as removal of a lead implanted for >12 months or requiring specialized tools) [5]. Understanding the specific subgroups of patients at high risk for complications during and after lead extraction has become imperative to properly manage endovascular CIED leads. There have been multiple published studies describing clinical variables that predict adverse outcomes in CIED system extractions [6–9]. In review of these large retrospective registries, the risk of complications in the cardiac transplant population has not been specifically addressed to date. Based on Organ Procurement and Transplantation Network data as of November 25, 2018, there were 3,242 cardiac transplants performed in the United States in 2017. It is estimated that 10.9% of transplant recipients develop bradyarrhythmias requiring pacemaker implantation, with the most common indication being sinoatrial dysfunction [10, 11]. We present four cases of transvenous extraction and removal of pacing leads implanted after cardiac transplantation.

## 2. Case One

A 75-year-old man with a past medical history of ischemic cardiomyopathy who underwent orthotopic heart transplantation (OHT) in 1997 (biatrial anastomosis) was referred for pacemaker system extraction. His initial posttransplant course had been complicated by sinus node dysfunction with a slow junctional escape rhythm, and he underwent implantation of a single chamber AAI Medtronic 8088B pacemaker with a Medtronic 4068 lead placed in the right atrium shortly after his transplantation. In 2007, the atrial lead had low impedance and impending failure, so a Medtronic 3830 lead was added in the right atrial appendage at the time of generator change.

He developed end-stage renal disease (ESRD) secondary to calcineurin inhibitor toxicity, and hemodialysis was started in 2012. He developed recurrent infections in his left upper extremity fistula site (initially methicillin-sensitive *Staphylococcus aureus* but later polymicrobial) in 2016 with eventual pacemaker pocket infection requiring full CIED system extraction.

The Medtronic 3830 lead, which had been indwelling for nine years, was extracted using laser energy application along the proximal portion of the lead. The older Medtronic 4068 lead, indwelling for 19 years, required extensive application of laser energy at multiple points along the lead for removal. The pocket was debrided, and the incision was closed using vertical mattress sutures. There was no temporary pacemaker placed, as he was not pacemaker-dependent. The patient was readmitted within 30 days due to concern that the pacemaker pocket site infection had not been fully cleared. This was ultimately treated by drainage of a complex fluid collection associated with the previous pacemaker site. The patient was admitted six months later due to sepsis secondary to disseminated histoplasmosis and ultimately died secondary to multiorgan failure.

## 3. Case Two

A 59-year-old man with a past medical history of nonischemic cardiomyopathy who initially underwent OHT in 1994 (biatrial anastomosis) was referred for pacemaker lead revision. His posttransplant course had been complicated by transplant vasculopathy, and he ultimately required a second heart transplant in 2002 (bicaval anastomosis). He also developed ESRD and underwent deceased donor kidney transplantation in 2004. He developed ehrlichiosis in 2011 in addition to cryptococcal pneumonia and histoplasmosis requiring chronic treatment with antifungals. In 2013, he had syncope leading to a subarachnoid hemorrhage and was diagnosed with sinus node dysfunction in the setting of intermittent sinus bradycardia to less than 20 beats per minute. He underwent dual chamber pacemaker placement in 2013 (Medtronic ADDRL1) with a Medtronic 5076 lead in the ventricular position and a Medtronic 5592 lead placed in the right atrial appendage after an active fixation lead was deemed to be unstable.

He was admitted for volume overload three years later, and pacemaker interrogation revealed undersensing on the atrial channel due to a gradual P wave amplitude decrease from 4.7 mV at implant to ~0.4 mV, leading to asynchronous ventricular pacing and failure to recognize atrial arrhythmias. No change in lead position was detectable on chest X-ray. An atrial lead addition was planned. However, the left subclavian vein was occluded. He underwent extraction of the atrial lead to obtain venous access. A 12 French Spectranetics SLS II laser sheath was advanced over the lead, and minimal application of laser energy was used to free adhesions. Countertraction using a snare was also employed from the femoral vein. The lead was removed, and subclavian access was retained. A Medtronic 3830 lead was implanted in the right atrium. The patient tolerated the procedure well, and he had no complications within the next 30 days. However, he was admitted with cryptogenic encephalopathy two months later which was thought to be at least partially related to subclinical cirrhosis. He was ultimately discharged to inpatient hospice and died shortly thereafter.

## 4. Case Three

A 60-year-old man with a past medical history of nonischemic cardiomyopathy who underwent OHT in 1994 was referred for pacemaker extraction (biatrial anastomosis). His posttransplant course was complicated by sinus node dysfunction, and he underwent dual chamber pacemaker placement (Medtronic P1501) in 2008 with Medtronic 3830 leads in the right atrium and right ventricle. He also developed ESRD secondary to calcineurin inhibitor toxicity and underwent deceased donor kidney transplant in 2008. He was admitted with sepsis secondary to *Escherichia coli* in 2014, and a TEE during this admission demonstrated vegetations involving the pacemaker leads. He underwent extraction of the six-year-old system with manual traction alone. His hospital course was complicated by worsening renal graft function thought to be secondary to sepsis, which ultimately required reinitiation of dialysis. He was discharged to a rehabilitation facility with a plan for four weeks of intravenous ceftriaxone but was subsequently readmitted with recurrent sepsis secondary to *Escherichia coli* within 30 days. He was found to have a left atrial appendage thrombus (despite sinus rhythm). The source of his persistent *E. coli* bacteremia was unknown; however, it was hypothesized that the left atrial appendage thrombus could have been a nidus for recurrent infection. He was discharged on a 6-week course of meropenem with eventual clearance of the bacteremia and reimplantation of a dual chamber pacemaker 10 months later. He ultimately died three years later after a prolonged hospital stay related to ascending cholangitis and septic shock as well as hemorrhagic shock related to a spontaneous retroperitoneal hemorrhage.

## 5. Case Four

A 68-year-old man with a past medical history of ischemic cardiomyopathy who underwent OHT in 1991 (biatrial anastomosis) was referred for pacemaker lead revision. His course had been notable for paroxysmal atrial fibrillation and sinus node dysfunction developing 25 years after transplantation. He underwent dual chamber pacemaker placement in 2017 (Medtronic A2DR01) with a Medtronic 3830 atrial lead and Medtronic 5076 ventricular lead.

He was admitted for management of atrial fibrillation with rapid ventricular response four months after device implantation. Device interrogation during the admission showed undersensing on the atrial channel. He underwent revision of the atrial lead 8 months after the initial implantation with lead removal via manual traction and new lead placement on the posterior right atrial septum due to poor sensing and pacing thresholds elsewhere. He had no immediate complications and was discharged home the same day. He had no complications within 30 days after discharge. However, he died from complications of recurrent aspiration pneumonia unrelated to the procedure two months after lead revision.

## 6. Discussion

We present four cases of transvenous extraction/removal of CIED leads placed after cardiac transplantation. All four of these patients underwent extraction for reasons that are plausibly related to their posttransplant status: two related to infection and two due to poor sensing on the atrial channel. Overall, these patients seemed to have a similar or slightly reduced degree of lead binding and tissue in-growth, with a 19-year-old lead requiring significant manual sheath dissection and laser-assisted dissection. More recent leads were removed more easily, often with manual traction. The average procedure duration was 92 minutes (longest 120 minutes, shortest 72 minutes), in comparison to estimated average procedure duration of 135 minutes in the largest single-center registry (though this comparison is clearly confounded) [8]. While no conclusions can be made based on the limitations of this small case series, this does suggest that there is likely no major increase in procedural duration related to prior transplantation status. In addition, three out of four patients died within seven months of the procedure (the fourth lived 44 months), though none had a major complication from the procedure itself. Overall, this may suggest that nonprocedural factors predict high mortality in patients requiring lead extraction after heart transplant.

Several attempts have been made to identify high-risk groups for developing adverse outcomes after lead extraction [6–9]. Brunner et al. analyzed more than 5000 cases of transvenous lead extraction in a single center and identified multivariable predictors of major complications [8]. The risk factors for major periprocedural complications were cerebrovascular disease, ejection fraction less than or equal to 15%, lower platelet count, INR greater than or equal to 1.2, use of mechanical sheaths, and use of powered sheaths. This study also identified predictors of all-cause mortality within 30 days of lead extraction, including body mass index less than 25, ESRD, advanced NYHA functional class, lower hemoglobin, higher INR, lead extraction for infection, and extraction of a dual coil ICD lead. Cardiac transplantation was not considered in the patient characteristics in this study, nor was it considered in other studies that attempted to identify high-risk populations [6–9].

Several prior studies have shown longer lead implant duration to be associated with an increased need for powered or mechanical sheaths as well as an increase in procedural failure [12–15]. In our series, increased lead dwell time (up to 19 years) seemed to be associated with a proportional increase in lead adhesions, as would be expected in a nontransplant population. Of note, case 4 was the only patient that had leads removed within 12 months of initial implantation. The other extractions ranged between 3 and 19 years after initial implantation.

Lead extraction for infection has previously been cited as a risk factor for mortality at 30 days and at one year after lead extraction [8, 16]. In our series, we present two patients who required system extraction secondary to infections (one case due to pocket infection; the other due to lead vegetations and bacteremia). *Staphylococcus aureus* (as seen in case one) is a relatively frequent pathogen in CIED-related infections [17]. The presence of ESRD with an arteriovenous fistula that became infected seemed to be the initial inciting event for this patient. Gram-negative *Escherichia coli* bacteremia (as seen in case three) is a relatively uncommon cause of lead-related endocarditis, and it is likely that it was related to underlying immunosuppression [17]. Case one required readmission within 30 days due to sepsis secondary to a persistent fluid collection at the site of the previous pacing system. Case three had readmission for sepsis and *E. coli* bacteremia of unclear etiology. Pocket site infection after extraction is an uncommon scenario after extraction for infection and is presumably related to the posttransplant immunosuppressed state.

In theory, chronic rejection leading to progressive fibrosis after cardiac transplantation could be a mechanism of poor sensing and capture over time after CIED implantation. In addition, partial or complete dislodgement may occur over time due to frequent right heart catheterization and endomyocardial biopsy with unintentional force placed on endocardial leads. In the two patients without infection, the initial procedural plan for lead failure was for lead addition rather than extraction and replacement. Given the relatively short lifespan of the patients in our cohort, this seems like a reasonable strategy when feasible. However, given the increased incidence of superior vena cava syndrome in some OHT populations (younger patients, bicaval anastomosis), procedural decision-making should be individualized as increased lead burden is thought to be a risk factor for this entity [18–20].

The predominant surgical technique used for cardiac transplantation has changed over time from primarily biatrial to bicaval anastomosis. While this series is too small to detect any difference in safety, it is plausible that surgical technique may affect the likelihood of major perioperative complications. In addition, it is thought that patients with prior cardiac surgery (including transplant) may be protected against some major adverse events, such as hemodynamically significant venous lacerations, due to the presence of adhesions, though the largest available dataset did not detect a significant difference [8].

## 7. Conclusions

We describe four patients who underwent transvenous extraction/removal of CIED leads placed after cardiac transplantation. There were no major complications related to extraction in these four cases. However, in three of the four cases in our series, the patients died within one year of extraction (2, 3, 7, and 44 months, respectively). Small numbers and the retrospective nature of this series preclude a comparison of the degree of lead binding and procedural complexity. For the same reason, a determination cannot be made regarding the appropriateness and safety of lead extraction for various indications in the transplant population. While the etiology of death in these cases seemed to be unrelated to the extraction procedure, the indications for extraction (infection in the setting of immunosuppression and calcineurin-associated ESRD and poor sensing/capture possibly related to chronic rejection) likely contributed at least indirectly to the subsequent death.
